# Relationship of patellar tendon micromorphology with body anthropometrics and lower-limb range of motion in volleyball athletes

**DOI:** 10.1371/journal.pone.0356562

**Published:** 2026-08-17

**Authors:** Jia Liu, Ya-Hsin Chang, Lisa Noceti-DeWit, Kornelia Kulig

**Affiliations:** 1 School of Health and Rehabilitation Sciences, College of Medicine, The Ohio State University, Ohio, United States of America; 2 Division of Biokinesiology and Physical Therapy, University of Southern California, Los Angeles, California, United States of America; University College Dublin, IRELAND

## Abstract

**Context:**

Volleyball athletes frequently perform jump-landings, which can lead to structural changes in the patellar tendon. However, the relationship between intratendinous morphology, anthropometrics, and lower-limb range of motion (ROM) in athletes remains unclear, and the distal region of the patellar tendon is understudied.

**Objective:**

To investigate the relationship between proximal and distal patellar tendon morphology with body anthropometrics and lower limb ROM in volleyball athletes.

**Participants:**

A total of 132 elite-level volleyball athletes.

**Main outcome measure(s):**

Tendon thickness and intratendinous architecture (i.e., collagen organization, indexed using the peak spatial frequency radius, PSFR) were assessed with ultrasound at the proximal and distal patellar tendons. Anthropometrics included body mass, height, and waist-to-hip ratio. Lower limb ROM included ankle dorsiflexion (knee-to-wall), hip internal and external rotations, and hip extension (modified Thomas test). Single-leg squat was also tested.

**Results:**

Athletes with patellar tendinopathy (51.5%) showed lower PSFR (1.59  ±  0.32 vs. 1.73  ±  0.31, p  =  0.025) at the proximal tendon than athletes without patellar tendinopathy. Proximal tendon PSFR was negatively associated with years of play (p = 0.002). Distal tendon PSFR was positively associated with ankle dorsiflexion (p = 0.039) and hip internal rotation ROM (p = 0.031), and negatively associated with body weight (p = 0.008), body height (p = 0.030), and waist-to-hip ratio (p = 0.022). In athletes without tendinopathy, distal PSFR was also negatively associated with hip extension ROM (p < 0.05).

**Conclusions:**

Proximal tendon PSFR decreased with more years of play, whereas lower distal tendon PSFR was associated with reduced ankle and hip ROM, greater body weight and height, and a higher waist-to-hip ratio, with small-to-medium effect sizes. Future studies should examine a broader range of biomechanical factors that influence regional tendon micromorphology.

## Introduction

Volleyball athletes frequently perform jump-landings, which can lead to structural changes in the patellar tendon [1–4]. These structural alterations can be observed on ultrasound images not only as increased thickness, but more prominently as a loss of the typically banded appearance within the tendon body [2]. Loss of the banded appearance of the tendon image generally indicates disorganized collagen fiber alignment [3], which has been suggested to signal the development of patellar tendinopathy [4,5]. As such, there has been growing interest in understanding the factors that contribute to patellar tendon health in volleyball athletes.

Prior studies investigating risk factors for altered patellar tendon morphology in volleyball athletes have primarily focused on extrinsic factors, including participation in ball sports in youth athletes [6], habitual loading in young adults [7], and even lifelong side-specific loading [8]. However, intrinsic factors such as body anthropometrics and lower limb range of motion (ROM) may also contribute to altered patellar tendon morphology [9–13]. Previous studies have reported that taller, heavier athletes with greater waist girth are more likely to develop patellar tendinopathy [9,10], suggesting a potential association of body anthropometrics and patellar tendon morphology. Additionally, studies have shown that hip and ankle ROM are associated with patellar tendinopathy in athletes [11–13]. However, no studies have explicitly assessed the relationships between micromorphological traits of the patellar tendon and body anthropometrics and lower limb ROM in volleyball athletes.

Another gap in the literature is that investigations of patellar tendon morphology have primarily focused on the proximal region while overlooking the distal region [5,7,14,15]. The patellar tendon is not structurally homogeneous [16], and uniquely has two osteotendinous junctions. Both regions experience higher strain than the tendon mid-substance during loading [17], which may contribute to region-specific adaptations or pathology. Prior studies using finite element modeling have also demonstrated regional differences in stress concentration [16], supporting the need for separate analysis. The distal patellar tendon and its potential relationship with body anthropometrics and lower limb range of motion (ROM) in volleyball athletes remains largely understudied.

Therefore, the purpose of this study was to investigate the relationship between proximal and distal patellar tendon micromorphology with body anthropometrics and lower limb ROM in volleyball athletes. We hypothesized that taller, heavier athletes who have greater waist girth and exhibit reduced lower limb ROM would demonstrate altered internal tendon architecture at the proximal, distal, or both proximal and distal patellar tendons. Understanding the roles of anthropometrics and lower limb ROM, which can be assessed using reliable, easily implementable clinical measures employed in this study, will provide valuable insights into timely injury risk assessments.

### Materials and Methods

#### Participants.

We recruited 132 volleyball athletes (99 males and 33 females). Each athlete was required to be at least 18 years old and to be actively competing at the elite level in volleyball at the time of study participation, such as the National Collegiate Athletic Association (NCAA) Division I or Olympic level. Participants were recruited through the our professional network of athletes, trainers, and coaches, and the study was presented during team meetings. All participants provided written informed consent approved by the Institutional Review Board at the University of Southern California, and data were collected between 22/09/2006 and 23/05/2010.

### Personal interview

Skilled clinicians (certified athletic trainers or physical therapists) conducted the survey via in-person interview with each participant to document their age, sex, years of play, choice of lead leg, and history of musculoskeletal injuries, including patellar tendon pain. The lead leg was defined as the leg in which the foot contacted the ground first at the beginning of the takeoff [18]. Skilled sports medicine clinicians determined the presence of patellar tendinopathy by palpating the tendon and verified whether they had mechanical load-related anterior knee pain, localized within the borders of the tendon, in both legs. Participants who reported current or recent mechanical load-related anterior knee pain in the lead leg were classified as having patellar tendinopathy.

During the interview, participants also completed the Victorian Institute of Sport Assessment – Patella (VISA-P) questionnaire, which measures pain and activity restrictions, with a lower VISA-P score indicating more pain and activity restrictions [19].

### Anthropometrics

We collected anthropometric data of body mass, body height, waist circumference, and hip circumference. Waist circumference was measured with tape around the waist at the narrowest level while the participant stood up straight and exhaled [20]. The hip circumference was measured at the broadest pelvic circumference [20]. We also calculated the waist-to-hip ratio using the waist circumference divided by the hip circumference, which has both excellent inter-observer and intra-observer reliabilities [20].

### Patellar tendon morphology

**Fig 1:**
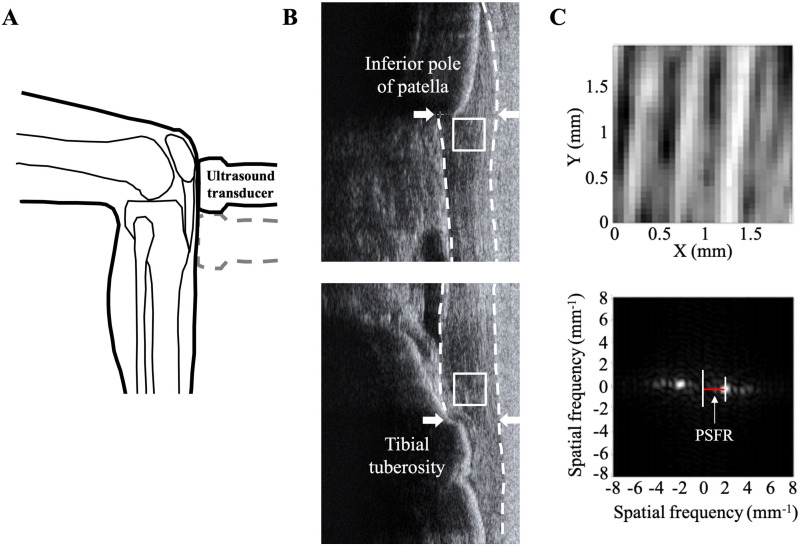
Ultrasound image acquisition and analysis for tendon morphology. (A) A schematic presentation of ultrasound transducer placements on the proximal (black solid line) and distal (gray dashed line) regions of the patellar tendon. (B) Representative ultrasound images of the patellar tendon at the proximal (top image) and distal (bottom image) regions. Patellar tendon border is outlined by the white dashed line. Tendon thickness was measured at the sites pointed by the white arrows: at the inferior pole of patella for the proximal tendon and at the proximal end of tibial tuberosity for the distal tendon. The selected regions of interest (ROIs, white boxes) are analyzed for micromorphology. (C) Top image is a zoomed-in view of a kernel within the ROI of a typical tendon. The red line in the image below represents the peak spatial frequency radius (PSFR) obtained through the corresponding frequency spectrum from the image above.

We measured patellar tendon morphology in the lead leg of each participant. Participants were seated comfortably on a hi-lo treatment table, elevated so that their feet did not touch the ground, allowing the lower limbs to hang freely. This gravity-dependent position helps provide a more consistent passive load to the patellar tendon and avoids the slackening often observed when the tendon is imaged in a knee-flexed position. Two experienced physical therapists and sonographers (K.K. & Y.C.) captured longitudinal B-mode ultrasound images of the patellar tendons using a commercial ultrasound scanner (Sonoline Antares, Siemens Medical Solutions USA Inc., Malvern, PA, USA) equipped with a linear probe (VFX 13−5, 4.5 cm aperture width, 10 MHz center frequency, 3.0 cm depth, 1.0 cm and 1.5 cm transmit foci). Images were captured in the long axis. The probe was placed at the inferior pole of the patella with the notch pointing proximally to image the proximal tendon, and at the point immediately proximal to the tibial tuberosity with the notch also pointing proximally to image the distal tendon (Fig 1A). Care was taken to ensure optimal quality and minimal movement artifacts.

For macromorphological analyses, tendon images were imported into ImageJ software (National Institute of Health, v.1.366, Bethesda, MD). We measured tendon thickness in the anteroposterior dimension at the inferior pole of the patella for the proximal thickness (Fig 1B). Distal tendon thickness was measured at the proximal end of the tibial tuberosity where the patellar tendon inserts (Fig 1B). We measured tendon micromorphology using previously published methods [2]. Regions of interest (ROIs) (Fig 1B) on tendon images were selected to analyze tertiary collagen fiber bundle organization [21] using MATLAB (MathWorks, Natick, MA). Polygonal ROIs immediately below the inferior pole of the patella and the area immediately proximal to the tibial tuberosity were selected for the proximal and distal tendon regions, respectively. The coordinates of the selected polygon points were recorded. Within the polygonal ROI boundary, every possible square kernel measuring 2 mm × 2 mm (32 by 32 pixels) was extracted and processed (Fig 1C). There was no down-sampling. The tendon boundaries were ignored to avoid edge artifacts. A two-dimensional (2-D) Fast Fourier Transform (FFT) was performed for each kernel within the selected ROI, and the spatial frequency parameters were calculated from all kernels and averaged. We have previously described the selection of spatial frequency parameters that capture tendon fiber organization [2], and the peak spatial frequency radius (PSFR) was chosen in this study. The PSFR is the distance from the spectral origin to the spatial frequency peak of greatest amplitude on the 2-D FFT spectrum (Fig 1C bottom image). It indicates the sparseness of the striated speckle pattern of the tendon on an ultrasound image (Fig 1C top image). Thus, a higher PSFR value indicates a tighter, more compact striated speckle pattern, which may correspond to better organization of the collagen fibers [3].

### Lower limb ROM and single-leg squat

We assessed each participant's lower limb ROM of the lead leg, including ankle dorsiflexion, hip internal and external rotations, and hip extension. Additionally, we evaluated active lower limb kinetic chain using a single-leg squat.

**Fig 2:**
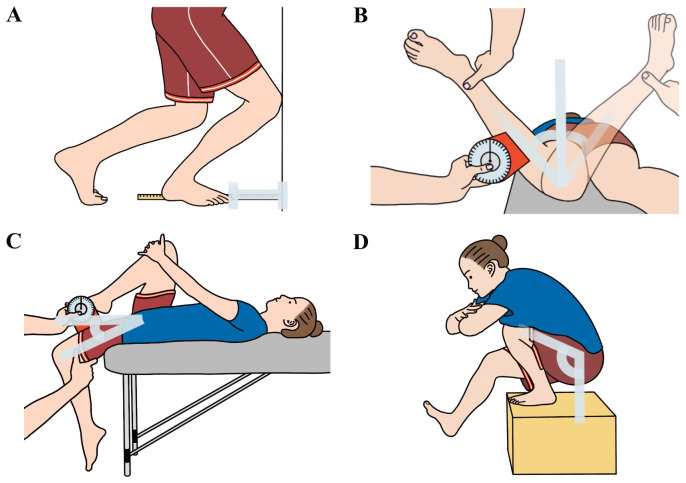
Measurements of lower limb range of motion and single-leg squat tests. (A) Ankle dorsiflexion was assessed using the distance (cm) between the wall and the most distal tip of the hallux during knee-to-wall. (B) Passive hip internal and external rotation were measured in angle (degree) between the vertical line and the tibia in a prone position. (C) Hip extension range of motion in the modified Thomas test was recorded as the angle (degree) of the thigh to the horizontal. (D) Single-leg squat was performed with the angle (degree) between the thigh and the vertical line measured at the lowest position in a successful trial.

At the ankle joint, dorsiflexion was measured using the knee-to-wall distance, a reliable test to assess functional mobility available at the ankle [22]. The participant stood and bent the testing ankle until the knee touched the wall, keeping the heel on the floor (Fig 2A). The distance between the wall and the most distal tip of the hallux was measured in centimeters. A larger distance indicates greater composite ankle dorsiflexion.

At the hip joint, the passive ROM for hip internal and external rotations was measured. Participants were instructed to lie prone on a treatment table with the knee of the testing leg flexed to 90 degrees [23] (Fig 2B). The examiner slowly and passively moved the vertical tibia outward to assess hip internal rotation and inward to assess hip external rotation ROM. During testing, care was taken to ensure that the pelvis and lumbar spine remained motionless. Upon sensing any pelvic movement, the examiner stopped the passive rotation and placed one arm of the inclinometer perpendicular to the table surface and the other arm along the tibia. The angle between the two arms of the inclinometer was measured, with a larger angle indicating a greater joint rotation (Fig 2B).

We also assessed hip extension ROM using the modified Thomas test. Participants lay supine on the treatment table with their thighs off the edge. The tested knee was maintained in 90 degrees of flexion, and the other knee was brought to the chest by the participant or examiner. Hip extension was measured using an inclinometer placed on the thigh, with the angle reported about the horizontal line (Fig 2C).

Besides passive ROM assessments, we evaluated a single-leg squat test, which combines flexibility, mobility, strength and balance and is a test for the entire kinetic chain and performance. The participant began in a single-leg standing position on a raised platform (46 cm), with arms crossed against their chest. The participant then squatted as low as possible while keeping the opposite leg in front. A successful single-leg squat required the participant to return to the initial single-leg standing position without losing balance from the lowest squatting point. At the lowest point of the squat, an inclinometer was used to measure the angle between the thigh and the vertical line in degrees (Fig 2D). A higher angular value indicates a deeper squat and better performance.

### Statistical analysis

The Shapiro-Wilk test was used to assess the normality of each variable of interest. For each variable of interest, including the PSFR and thickness at the distal and proximal regions of the patellar tendon, VISA-P score, years of play, age, body weight, body height, waist-to-hip ratio, knee-to-wall distance, range of hip internal rotation, range of hip external rotation, range of hip extension, and the single-leg squat angle, group differences between athletes with and without patellar tendinopathy were analyzed using multiple linear regression models with the presence of patellar tendinopathy as the primary independent variable. To account for potential confounding by playing positions, all models were adjusted for athletes’ primary playing position (libero, outside hitter, opposite hitter, middle blocker, or setter) as a covariate.

Multiple linear regression was used to examine the relationship between tendon micromorphology (i.e., PSFR) and each anthropometric and lower limb measure, with separate models for each independent variable. In each model, playing position (libero, outside hitter, opposite hitter, middle blocker, or setter) was included as a covariate to account for positional differences in body composition and playing demands. To determine whether the presence of patellar tendinopathy confounded or moderated the relationship between each independent variable and PSFR, an interaction term between patellar tendinopathy presence and each independent variable was included. When no significant interaction was observed, the main-effects model was interpreted, with the regression coefficient reflecting the association between the independent variable and PSFR after adjusting for both playing position and tendinopathy presence. When a significant interaction was detected, the relationship was examined separately within each group using simple slopes analysis. We tested regression models for proximal and distal tendon PSFR separately. All statistical analyses were performed using R Statistical Software (version 4.2.0; R Foundation for Statistical Computing, Vienna, Austria), with a significance level α = 0.05.

## Results

Of the 132 athletes studied, 64 (48.5%) were asymptomatic and 68 (51.5%) were classified as having patellar tendinopathy. In athletes with patellar tendinopathy, pain was confined to the proximal patellar tendon, with no reports of distal patellar tendon pain.

Descriptive results for each variable of interest are presented in Table 1. Of all athletes studied, 30 were active Olympic-level athletes and the remaining 102 were NCAA Division I collegiate athletes. Athlete positions included 13 liberos, 49 outside hitters, 16 opposite hitters, 33 middle blockers, and 21 setters. After adjusting for playing position, athletes with patellar tendinopathy had significantly lower VISA-P scores (69.0 ± 16.2 vs. 82.6 ± 15.7, p < 0.001), lower PSFR (1.59 ± 0.32 vs. 1.73 ± 0.31, p = 0.025) at the proximal region of the patellar tendon, and greater body weight (88.3 ± 9.9 kg vs 83.3 ± 11.5 kg, p = 0.028), when compared to athletes without patellar tendinopathy. However, no significant between-group differences were found in tendon thickness, distal tendon morphology, other anthropometrics, or lower limb variables (p > 0.05).

**Table 1:** Participant demographics and characteristics of anthropometrics, lower limb range of motion, and tendon morphology.

	Patellar Tendinopathy (n = 68)			No Patellar Tendinopathy (n = 64)			All Participants (N = 132)		
	Mean ± SD	Range	Number of participants	Mean ± SD	Range	Number of participants	Mean ± SD	Range	Number of participants
**Demographics and Body Morphometrics**									
Sex (male / female)	–	–	56 / 12	–	–	43 / 21	–	–	99 / 33
Height (cm)	192.7 ± 10.1	163.8 – 208.3	68	188.8 ± 9.7	160.6 – 210.8	64	190.8 ± 10.1	160.6 – 210.8	132
Male	194.9 ± 9.0	165.1 – 208.3	56	192.6 ± 7.9	175.3 – 210.8	43	193.9 ± 8.5	165.1 – 210.8	99
Female	182.3 ± 9.1	163.8 – 191.8	12	181.1 ± 8.3	160.6 – 193.0	21	181.5 ± 8.5	160.6 – 193.0	33
Weight (kg)	88.3 ± 9.9	67.8 – 113.4	68	83.3 ± 11.5	56.8 – 113.3	64	85.9 ± 11.0	56.8 – 113.4	132
Male	90.5 ± 9.2	70.8 – 113.4	56	88.5 ± 9.0	69.0 – 113.3	43	89.6 ± 9.1	69.0 – 113.4	99
Female	78.1 ± 6.4	67.8 – 91.6	12	72.6 ± 8.3	56.8 – 89.9	21	74.6 ± 8.0	56.8 – 91.6	33
Years of play (years)	9.5 ± 3.8	3.0 – 20.0	68	9.8 ± 3.3	3.0 – 21.0	59	9.7 ± 3.5	3.0 – 21.0	127
Male	9.05 ± 3.7	3.0 – 20.0	56	9.1 ± 3.4	3.0 – 21.0	39	9.1 ± 3.5	3.0 – 21.0	95
Female	11.6 ± 3.8	6.0 – 20.0	12	11.15 ± 2.6	6.0 – 16.0	20	11.3 ± 3.1	6.0 – 20.0	32
Waist-to-hip ratio*^a^*	0.81 ± 0.04	0.71 – 0.95	68	0.81 ± 0.05	0.7 – 0.9	60	0.81 ± 0.05	0.70 – 0.95	128
VISA-P score (0-100**)*^b^*	69.0 ± 16.2	31.0 – 100.0	65	82.6 ± 15.7	45.0 – 100.0	58	75.4 ± 17.3	31.0 – 100.0	123
**Lower Limb Range of Motion**									
Knee-to-wall distance (cm)*^c^*	12.3 ± 3.6	4.5 – 19.0	68	12.5 ± 3.6	2.0 – 24.0	60	12.4 ± 3.6	2.0 – 24.0	128
Hip internal rotation (degrees)	30.6 ± 10.8	3.0 – 58.0	68	32.1 ± 9.0	13.0 – 62.0	60	31.3 ± 10.0	3.0 – 62.0	128
Hip external rotation (degrees)	38.6 ± 11.6	3.0 – 62.0	68	37.0 ± 10.2	18.0 – 60.0	60	37.8 ± 11.0	3.0 – 62.0	128
Hip extension (modified Thomas test with knee flexed, degrees)	20.6 ± 6.3	8.0 – 35.0	68	19.8 ± 6.9	5.0 – 35.0	60	20.2 ± 6.6	5.0 – 35.0	128
Single-leg squat (degrees)*^d^*	63.4 ± 27.2	10.0 – 138.0	68	61.3 ± 23.2	28.0 – 120.0	59	62.4 ± 25.3	10.0 – 138.0	127
**Patellar Tendon Morphology**									
Proximal tendon thickness (mm)	5.25 ± 1.17	3.22 – 7.86	56	4.83 ± 0.86	3.30 – 7.48	46	5.06 ± 1.06	3.22 – 7.86	102
Proximal tendon PSFR (mm^-1^)*^e^*	1.59 ± 0.32	0.94 – 2.32	55	1.73 ± 0.31	1.0 – 2.41	42	1.65 ± 0.32	0.94 – 2.41	97
Distal tendon thickness (mm)	4.91 ± 0.94	2.95 – 7.59	56	4.65 ± 0.87	3.11 – 6.68	46	4.80 ± 0.91	2.95 – 7.59	102
Distal tendon PSFR (mm^-1^)*^e^*	1.62 ± 0.30	1.11 – 2.70	56	1.71 ± 0.31	0.96 – 2.46	43	1.66 ± 0.31	0.96 – 2.70	99

Data in the table are presented as mean ± standard deviation (SD), range, and number of participants for whom data were available.

^*a*^Waist-to-hip ratio: a higher value suggests more body mass distributed at the waist, i.e., an android body shape.

^^b^^VISA-P, Victorian Institute of Sport Assessment – Patella: ** score ranges from 0 to 100, with a higher score indicating less pain and better function.

^*c*^Knee-to-wall distance: a higher number indicates greater ankle dorsiflexion mobility.

^*d*^Single-leg squat: a higher value indicates a deeper squat.

^*e*^PSFR, peak spatial frequency radius: a measure of tertiary collagen bundle organization (micromorphology), with higher values indicating better collagen fiber organization in tendon morphology.

Weight (p < 0.001) and height (p < 0.001) differed significantly across playing positions. Liberos were the lightest and shortest (73.9 ± 9.3 kg and 1.74 ± 0.08 m, all pairwise p ≤ .037), while opposite hitters (94.8 ± 11.7 kg, 1.96 ± 0.07 m) and middle blockers (88.0 ± 10.1 kg, 1.97 ± 0.07 m) were the tallest and heaviest, with setters (83.9 ± 11.9 kg, and 1.86 ± 0.09 m) outside hitters (85.3 ± 8.1 kg, 1.92 ± 0.07 m) falling in between. No other variable of interest differed by position (all p > 0.10).

### Variables related to PSFR at the proximal region of the patellar tendon

**Fig 3:**
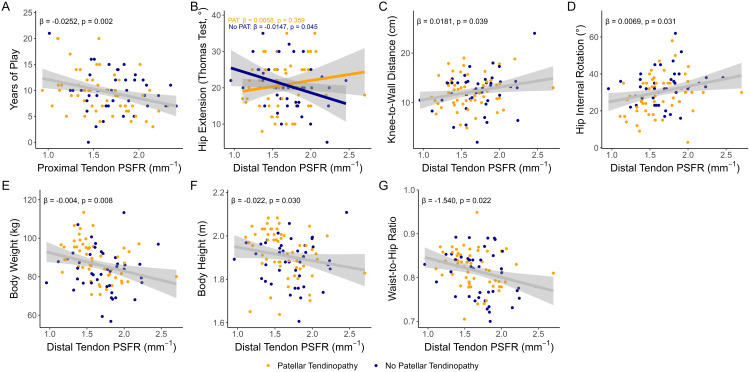
Associations between patellar tendon peak spatial frequency radius (PSFR) and significant predictors identified in regression models adjusted for playing position and patellar tendinopathy presence. (A) Years of play and proximal tendon PSFR. (B) Hip extension range of motion (Thomas Test) and distal tendon PSFR, with separate regression lines for athletes with and without patellar tendinopathy, reflecting a significant interaction (p = 0.035). (C) Knee-to-wall distance and distal tendon PSFR. (D) Hip internal rotation range of motion and distal tendon PSFR. (E) Body weight and distal tendon PSFR. (F) Body height and distal tendon PSFR. (G) Waist-to-hip ratio and distal tendon PSFR. Scatter plots display bivariate relationships for visualization; reported β coefficients and p-values are derived from adjusted regression models. Effect sizes ranged from small to medium (η²p = 0.05–0.10). PAT: patellar tendinopathy**.**

No significant interactions between patellar tendinopathy presence and any independent variable were observed for proximal tendon PSFR (all p > 0.05), indicating that the relationships between each variable and proximal tendon PSFR did not differ between athletes with and without patellar tendinopathy. In the main-effects models, after adjusting for playing position and patellar tendinopathy presence, years of play were negatively associated with proximal tendon PSFR (β = −0.025, p = 0.002, η²_p_ = 0.10, Fig 3A), indicating that athletes with more years of play had lower proximal tendon PSFR values. No other variables of interest were significantly associated with proximal tendon PSFR (all p > 0.05, S1 Fig). Playing position, included as a covariate, was not a significant predictor of proximal tendon PSFR in any model (all p > 0.05).

### Variables related to PSFR at the distal region of the patellar tendon

A significant interaction was observed between the hip extension and patellar tendinopathy presence for distal tendon PSFR (p = 0.035), indicating that the relationship between hip extension and distal tendon PSFR differed between groups. Simple slopes analysis revealed that greater hip extension ROM was associated with lower distal tendon PSFR in athletes without patellar tendinopathy (β = −0.015, p < 0.05, η²p = 0.05, Fig 3B). In contrast, no significant association was observed in athletes with patellar tendinopathy (β = 0.006, p > 0.05). For all remaining variables, no significant interactions were detected (all p > 0.06), and main-effects models were interpreted. After adjusting for playing position and patellar tendinopathy presence, distal tendon PSFR was positively associated with knee-to-wall angle (β = 0.018, p = 0.039, η²_p_ = 0.05, Fig 3C) and hip internal rotation ROM (β = 0.007, p = 0.031, η²_p_ = 0.05, Fig 3D), and negatively associated with body weight (β = −0.004, p = 0.008, η²_p_ = 0.07, Fig 3E), body height (β = −0.022, p = 0.030, η²_p_ = 0.05, Fig 3F), and waist-to-hip ratio (β = −1.54, p = 0.022, η²_p_ = 0.06, Fig 3G). No other variables were significantly associated with distal tendon PSFR (all p > 0.05, Supplementary Fig. 1). Playing position, included as a covariate, was not a significant predictor of distal tendon PSFR in any model (omnibus p > 0.05).

## Discussion

This study aimed to characterize the relationship between proximal and distal patellar tendon micromorphology with body anthropometrics and lower limb ROM in elite volleyball athletes. Consistent with the literature, we found that athletes with patellar tendinopathy exhibited signs of local tendon degeneration at the proximal region, including disorganized collagen fibers (i.e., lower PSFR values), particularly in those with more years of play. For the first time, we also revealed that, while distal tendon micromorphology did not differ between athletes with and without patellar tendinopathy, lower PSFR was associated with greater body mass, taller stature, higher waist-to-hip ratio, and reduced hip and ankle joint ROM, independent of tendinopathy status. These associations were small to medium in effect size, suggesting that intrinsic body factors may contribute modestly to variation in tendon micromorphology. Our results provided novel insights into patellar tendon morphology in volleyball athletes, while highlighting the need for future studies to examine a broader range of biomechanical factors.

The athletes in this study represent typical elite-level, active volleyball athletes [24,25]. Among them, 51.5% experienced patellar tendinopathy, with pain localized to the proximal region of the tendon. This finding aligns with previous reports indicating that 40–50% of volleyball athletes experience symptoms related to patellar tendinopathy [24,25].

Our results revealed that volleyball athletes with patellar tendinopathy showed signs of tendon degeneration at the proximal region, characterized by lower PSFR values. Lower PSFR values suggest disorganized collagen fiber alignment [3] and degraded properties of tendon stiffness and elastic modulus [26]. Poor collagen organization, coupled with increased collagen type III production, contributes to tendon thickening, indicating degeneration [27]. Our results agree with previous findings that tendinopathic tendons are more disorganized with less dense structural alignment [4,28,29]. Tendon degeneration has been attributed to overloading of the patellar tendon during athletic activities [27]. Supporting this, our results revealed that athletes with more years of play, likely accumulating more jumps, had lower PSFR values at the proximal patellar tendon.

After adjusting for playing position and patellar tendinopathy presence, distal tendon PSFR showed modest associations with hip and ankle joint ROM (Fig 3C, 3D), with small-to-medium effect sizes (η²p = 0.05). Athletes with poorer collagen fiber organization (i.e., lower PSFR value) in the distal patellar tendon exhibited reduced hip internal rotation and ankle dorsiflexion. Our results are in line with findings of Omodaka et al., who investigated lower limb ROM in children and adolescent athletes with Osgood-Schlatter disease, characterized by pain at the distal patellar tendon attachment to the tibial tuberosity [30]. Omodaka et al. found that children and adolescent athletes with symptomatic Osgood-Schlatter disease exhibited significantly reduced hip internal rotation and ankle dorsiflexion ROM, whereas asymptomatic participants with the condition showed non-significant but greater lower limb tightness than controls [30]. Our post-hoc analyses revealed that 25% of the athletes in the current study recalled experiencing pain at the tibial tuberosity in childhood and adolescence, and some of them remembered being diagnosed with Osgood-Schlatter disease, even though such pain subsided beyond adolescence. However, our participants with and without a history of adolescent distal patellar tendon pain showed no significant differences in tendon morphology, anthropometrics, and lower limb ROM. The statistical discrepancy between our findings and those of Omodaka et al.’s may be due to differences in participant age at the time of data acquisition and the fact that we relied on athletes’ recall regarding the presence of Osgood-Schlatter disease [30]. Nevertheless, findings from collegiate athletes suggest that reduced hip and ankle joint ROM may be linked to a history of adolescent pain at the distal patellar tendon.

In addition, we found that among athletes without patellar tendinopathy, those with more disorganized collagen fibers in the distal patellar tendon showed greater hip extension ROM. Our results suggest that greater hip joint ROM may affect movement control strategies, potentially placing greater strain on the knee, which may be associated with worse tendon morphology. From the tissue biomechanics perspective, an alternative explanation is that the patellar tendon may act as a primary restraint to hip extension during the modified Thomas test, and that tendinopathic tendons may exhibit greater compliance, thereby reducing restraint. However, no studies have demonstrated that the patellar tendon or quadriceps muscles are the primary restraint to hip extension in this position. Future studies should consider investigating the direct relationship between patellar tendon properties and hip extension.

We also observed significant relationships between distal tendon micromorphology with body weight and waist-to-hip ratio (Fig 3E, 3G). Higher body weights and greater waist-to-hip ratios suggest android fat distribution, which is associated with preclinical systemic diseases, such as diabetes, elevated serum lipids, and hyperuricemia [31]. These systemic diseases may, in turn, alter collagen fiber properties and have been linked to a higher prevalence of degenerative tendinopathies [32]. Supporting this, a prior study on volleyball athletes found that those with patellar tendinopathy had greater waist-to-hip ratios [9].

This study has several limitations. First, our sample consisted solely of elite volleyball players currently competing. Thus, caution is warranted when generalizing our results to recreational players or those with severe tendinopathy who have retired. Second, our cohort was predominantly male, although we did not find significant sex differences in the likelihood of patellar tendinopathy. Third, we examined the active lower limb kinetic chain using a single-leg squat test. Future studies could incorporate a broader range of dynamic assessments to further explore the relationship between tendon morphology and athletic performance. Fourth, symptom status may vary depending on training load prior to data collection, and elite athletes may underreport symptoms to protect their competitive standing. As such, we were unable to reliably distinguish between currently symptomatic and asymptomatic athletes at the time of data collection.

Our study showed that, in the current cohort of college and elite volleyball athletes, disorganized distal tendon collagen fibers were associated with higher body weight and greater waist fat accumulation. That body type has been linked to a predisposition to, or a precursor of, metabolic dysregulation. Interestingly, greater disorganization of distal tendon collagen fibers was also associated with reduced range of motion at the hip and ankle joints in collegiate athletes. Overall, our novel findings highlight relationships among body anthropometrics, lower limb ROM, and distal tendon micromorphology, underscoring the importance of considering both systemic and mechanical perspectives on tendon health.

## Supporting information

S1 Fig Non-significant associations between patellar tendon peak spatial frequency radius (PSFR) and variables of interest, from regression models adjusted for playing position and patellar tendinopathy presence. Panels A–I show proximal tendon PSFR: (A) VISA-P score, (B) body weight, (C) body height, (D) waist-to-hip ratio, (E) knee-to-wall distance, (F) hip internal rotation range of motion, (G) hip external rotation range of motion, (H) hip extension range of motion (Thomas Test), and (I) single-leg squat angle. Panels J–M show distal tendon PSFR: (J) VISA-P score, (K) years of play, (L) hip external rotation range of motion, and (M) single-leg squat angle. None of these associations reached statistical significance after adjusting for playing position and patellar tendinopathy presence (all p > 0.05). Scatter plots display bivariate relationships for visualization; reported β coefficients and p-values are derived from adjusted regression models.
(TIFF)
