# Supplementary material for: Relationship of patellar tendon micromorphology with body anthropometrics and lower-limb range of motion in volleyball athletes
Source: PLoS One. 2026 Aug 17;21(8):e0356562. doi: 10.1371/journal.pone.0356562 (PMC13480626; doi:10.1371/journal.pone.0356562)
Supplement: Supplementary file 1 [file pone.0356562.s003.docx]

We thank the reviewers for their thoughtful and constructive comments, which have helped us improve the clarity and rigor of our manuscript. We have provided a point-by-point response to each comment below and hope that the revisions adequately address the concerns raised. All changes to the manuscript are highlighted in the revised version for ease of reference.

Reviewer #1: I read this manuscript and I enjoyed the concept of the research which focuses on sports injuries and the wellbeing of tendons. There are many points to address before the manuscript is ready for publication.

Explain how the participants were made aware of this study and thus recruited in it.
It is important in a study like this to split volleyball players according to their playing position. A libero does not jump as much as attacking and blocking players, and setters don’t have the same jump and land biomechanics as hitters. Therefore, the authors must correct for playing position: liberos, setters, and attacking players. Also, it would be very interesting to see if the different players would show different patellar anatomies.

**Response re. Recruitment:** We thank the reviewer for this important comment. Using our professional network with athletes, trainers, and coaches, we presented the study to the athletes during one of their team meetings. Additional information was provided using flyers. Participation was voluntary. As an incentive, we indicated that the individual who interpreted the data will be available to the athlete for a consultation. We have added to the manuscript (page 5) the following text: “Participants were recruited through the our professional network of athletes, trainers, and coaches, and the study was presented during team meetings.”

**Response re. Playing position**: Yes, the number of jumps varies between players during a game. A quick search resulted in the following number of jumps per game: middle blockers (18-23), setters (17-22), opposite hitters (15-19), outside hitters (12-16). There is also the issue of jump height, which contributes to the load on the musculoskeletal system. While our study was designed to examine tendon morphology in volleyball athletes broadly rather than to compare positions, we appreciate the reviewer's suggestion and have addressed it in two ways.

First, we included playing position (libero, outside hitter, opposite hitter, middle blocker, or setter) as a covariate in all regression models to account for potential confounding. Rather than excluding liberos, we retained all athletes and statistically adjusted for positional differences to preserve statistical power and generalizability.

Second, we conducted exploratory analyses to assess whether patellar tendon anatomy differed across positions. Body weight (p < 0.001) and height (p < 0.001) differ significantly across playing positions. Liberos were the lightest and shortest (73.9 ± 9.3 kg, 1.74 ± 0.08 m; all pairwise p ≤ 0.037), whereas opposite hitters (94.8 ± 11.7 kg, 1.96 ± 0.07 m) and middle blockers (88.0 ± 10.1 kg, 1.97 ± 0.07 m) were the tallest and heaviest, with outside hitters (85.3 ± 8.1 kg, 1.92 ± 0.07 m) and setters (83.9 ± 11.9 kg, 1.86 ± 0.09 m) falling in between. However, playing position was not a significant predictor of PSFR at either the proximal (p = 0.89) or distal (p > 0.11) tendon region, nor did it significantly affect any other variable of interest (all p > 0.10). These results indicate that, despite anthropometric differences between positions, patellar tendon micromorphology did not vary by playing position in our cohort.

The revised methods and results sections now reflect these analyses (pages 11–15).

Please describe the breakdown between D1 and Olympic players. I noticed that the age range goes up to 34. was that specific athlete still playing professionally at that time, or have they retired and now play recreationally while maintaining the same workout regimen and play time?

**Response:** We thank the reviewer for this question. Of the total sample, 30 participants were Olympic-level athletes who were actively playing professionally at the time of the study. The remaining participants were Division I collegiate players. This breakdown has been clarified in the revised manuscript (page 12)

Regarding the oldest participant (age 34), this individual was indeed still playing professionally at the time of data collection. This athlete represented the United States national team from 1996 to 2009 and was actively competing at the professional level when enrolled in the study. No retired or recreational players were included in the sample.

It is not clear how many players never had PT, how many have a history but were asymptomatic when they participated in the study. This is important to show.

**Response:** We thank the reviewer for raising this important point. All participants were actively competing and training at the time of the study. We recognize that elite athletes are often reluctant to disclose current pain, particularly out of concern that it may affect their being considered for competing. For this reason, rather than asking about current symptoms, we asked participants whether they had a recent history of patellar tendon pain around the season, which we believed would elicit more candid responses. The breakdown of participants with a history of patellar tendinopathy but who were asymptomatic at the time of testing is unknown. We have also acknowledged the potential for biased or underreported symptom status as a limitation of the study (page 18).

Table1 needs to be split between no history of PT, history of PT but currently asymptomatic, and the currently symptomatic PT group.

**Response:** We appreciate this suggestion. However, as noted above, because elite athletes are reluctant to disclose current symptoms for fear that doing so may affect their consideration for competition, we collected a history of patellar tendon pain rather than current symptom status. As such, we are unable to distinguish between currently asymptomatic and currently symptomatic participants. Table 1 has been revised to stratify participants by history of patellar tendinopathy versus no history (page 13). We have acknowledged the inability to further classify current symptom status as a limitation of the study.

Knowing that pain is poorly related to tendon morphology, we focused this analysis and this manuscript on the relationship between tendon morphology and ROM and anthropometrics.

Table1 Years of play: the range starts from 0...how is someone with 0 years of experience playing NCAA volleyball. Also, if they have 0 years of experience how are they suitable for inclusion in this study of elite players. Surely there should be a minimum number of years applied as inclusion criterion. How many players have less than 2 years experience?

**Response:** We thank the reviewer for identifying this discrepancy. The participant who reported 0 years of play was an athlete who had competed at the national level for less than half a year, which was rounded down to 0. We were unable to trace this participant to determine her total years of play across all competitive levels. We therefore treated her years-of-play data as missing, and the mean, standard deviation, and range in Table 1 have been recalculated accordingly (page 13). The minimum now reflects 3 years of play. All participants, including this individual, met the inclusion criteria of actively competing at the elite level (Division I or national/Olympic team) at the time of the study.

Also, here it is important to split males from females especially that females in the US start playing volleyball at a younger age than males. Therefore, this will affect the average years of play.

**Response:** We agree. Table 1 has been revised to present years of play stratified by sex (page 13).

Reviewer #2: The study is of interest and thorough, albeit carried out a long time ago (almost 20 years). Do the authors foresee any changes in the management of tendinopathy over the last 20 years affecting the relevance of the study today?

**Response:** We thank the reviewer for this excellent question. We consider these findings novel, eye-opening, and relevant to coaching and managing the health of jump-landing athletes. These findings remind us that a history of loading during developmental stages and overall health are relevant to the contemporary management of athletes. We have not seen an explicit acknowledgment of incorporating a history of Osgood-Schlatter’s disease into the management of adult jumping athletes with patellar tendinopathy. Therefore, we consider this work a call to the athletes’ care community to take a careful history and to incorporate the assumption of altered tendon structure into athlete care.

The authors set out their aim to understand how structural changes from jump-landings relate to anthropometrics and range of motion. Firstly, the authors replicate known results that tendinopathy correlates with increased tendon thickness and decreased PSFR. Because of this, the inclusion of participants both with and without tendinopathies is a clear confounding factor which makes it difficult to decouple whether the correlations observed relate to an individual's tendon morphology or their history of tendinopathy.

**Response:** We appreciate the reviewer's attention to this important methodological consideration. We agree that the inclusion of athletes with and without patellar tendinopathy requires careful analytical handling to decouple associations related to tendon morphology from those driven by tendinopathy history.

To rigorously address this concern, we used a two-step analytical approach. First, for each variable of interest, we fit a multiple linear regression model that included an interaction term between patellar tendinopathy status and the independent variable, with playing position as an additional covariate (per reviewer 1’s comment). The interaction term directly tests whether the relationship between each predictor and PSFR differs between athletes with and without patellar tendinopathy. When no significant interaction was detected (19 of 20 models), we interpreted the main-effects model, with the regression coefficient reflecting the association between the independent variable and PSFR after adjusting for both tendinopathy status and playing position. When a significant interaction was detected (Thomas Test × distal tendon PSFR, p = 0.035), we examined the relationship separately within each group using simple slopes analysis.

This transparent approach allows us to determine whether observed associations reflect tendon morphology broadly or are specific to one group, without excluding a meaningful portion of our elite athlete sample. Notably, for all significant associations at the distal tendon (body weight, height, waist-to-hip ratio, knee-to-wall angle, and hip internal rotation), no significant interactions with tendinopathy were observed, confirming that these relationships are independent of tendinopathy.

Throughout, very weak correlations are oversold based on the statistical significance of these weak correlations. I worry this will mislead readers as to the importance of these correlations. Regardless of correlation strength, publication of the results is certainly of interest to the field, but the weakness of these correlations should be made much clearer to readers throughout.

**Response:** We thank the reviewer for this important feedback and agree that the strength of the observed associations should be clearly communicated to readers. In our revised manuscript, we have made several changes to address this concern.

First, we replaced bivariate correlation analyses with multiple linear regression models that were adjusted for playing position and the presence of patellar tendinopathy. Rather than reporting correlation coefficients (r), we now report regression coefficients (β) with corresponding p-values. Second, we have added partial eta-squared (η²p) effect sizes for all significant associations to provide readers with a transparent measure of the variance explained by each predictor. Effect sizes ranged from 0.05 to 0.10, which we explicitly characterize as small to medium throughout the manuscript. Third, we have revised the language in the results, discussion, and conclusions to avoid overstating the importance of these associations. Finally, we now visualize all significant and non-significant associations with scatter plots (Figure 3 and Supplementary Figure 1, page 15 and page 20), enabling readers to judge the strength of the relationships for themselves.

We believe these changes provide an honest and transparent presentation of the data while preserving the novel contribution of these findings to the field.

e.g. "A novel finding of our study is that distal tendon micromorphology, as indexed by the PSFR, is significantly associated with hip and ankle joint ROM (Fig 3A)" - Figure 3A shows an incredibly low r^2 = 0.0484, and the tone of the text misrepresents this as an important correlation. Another example is in the conclusions: "Athletes with worse distal tendon morphology had reduced hip and ankle ROM, larger body mass, and greater mass concentration around the waist" - anyone can see from Figure 3 that this is hyperbole and does not reflect the reality of the results. Equally, I'm not convinced the title of the manuscript is supported.

**Response:** We thank the reviewer for these specific examples and agree that the original language overstated the strength of the observed associations. We have made substantive changes to address this concern.

The tone throughout the manuscript has been revised. For example, the sentence "A novel finding of our study is that distal tendon micromorphology, as indexed by the PSFR, is significantly associated with hip and ankle joint ROM" has been replaced with language that explicitly acknowledges the modest nature of these relationships. We now report partial eta-squared (η²p) effect sizes alongside all regression coefficients, which ranged from 0.05 to 0.10 (small to medium). In the discussion, we now state: "These associations were small to medium in effect size, suggesting that intrinsic body factors may contribute modestly to variation in tendon micromorphology" (page 16).

The conclusions in the abstract have been revised from "Athletes with worse distal tendon morphology had reduced hip and ankle ROM, larger body mass, and greater mass concentration around the waist" to: "Proximal tendon PSFR decreased with more years of play, while lower distal tendon PSFR was associated with reduced ankle and hip ROM, greater body weight and height, and higher waist-to-hip ratio, with small-to-medium effect sizes. Future studies should examine a broader range of biomechanical factors influencing regional tendon micromorphology" (page 2–3).

The title has been revised to: " Relationship of patellar tendon micromorphology with body anthropometrics and lower-limb range of motion in volleyball athletes" (page 1).

A majority of the results are merely stated without graphical representation of the data and, given the above issues, it would be in the reader's interest to have visualisation of all stated results, either in figures in the main article or in supplementary material.

**Response:** We agree and thank the reviewer for this suggestion. We have added graphical representations of all reported associations. Figure 3 now displays scatter plots for all significant associations (7 panels), including regression coefficients (β), p-values, and separate trend lines for the Thomas Test interaction. Additionally, Supplementary Figure 1 presents scatter plots of all non-significant associations (13 panels), along with the corresponding β and p-values from the adjusted regression models. Together, these visualizations enable readers to directly evaluate the strength, direction, and spread of all reported relationships (see figure legends on page 15 and page 20).

The introduction, methodology, and discussion sections mention a single-leg squat test, the results of which don't appear to be included.

**Response:** We thank the reviewer for noting this oversight. The single-leg squat angle was not significantly associated with PSFR at either the proximal (β = 0.0006, p = 0.651) or distal (β = −0.0003, p = 0.812) tendon region after adjusting for playing position and patellar tendinopathy presence. These results were inadvertently omitted from the original manuscript. In the revised version, single-leg squat results are now explicitly reported in the Supplementary Figure 1 (panels I and M).

The acquired data is worthy of publication, however as the current conclusions are not supported by the data, I cannot recommend publication in its current state.

**Response:** We thank the reviewer for recognizing the value of the data and for the constructive feedback that has strengthened our manuscript. We have made several substantive revisions to ensure that our conclusions are proportionate to the data. Specifically, we (1) replaced bivariate correlations with multiple linear regression models adjusted for playing position and the presence of patellar tendinopathy, (2) added partial eta-squared effect sizes for all significant associations and explicitly characterized them as small to medium (η²p = 0.05–0.10), (3) revised the language throughout the results, discussion, and conclusions to avoid overstating the strength or clinical importance of the observed relationships, (4) provided graphical visualizations of all associations (significant and non-significant) to enable readers to judge the data directly, and (5) reframed the conclusions to call for future studies examining a broader range of biomechanical factors. We believe these revisions align the manuscript's claims with the data, and we hope the reviewer finds the revised version suitable for publication.
