# Supplementary material for: Relationship of patellar tendon micromorphology with body anthropometrics and lower-limb range of motion in volleyball athletes
Source: PLoS One. 2026 Aug 17;21(8):e0356562. doi: 10.1371/journal.pone.0356562 (PMC13480626; doi:10.1371/journal.pone.0356562)
Supplement: Peer review history

# Peer review: Decision Letter 0

**DOI:** 10.1371/journal.pone.0356562.r001

**Authors:** Laura-Anne Marie Furlong

3 May 2026

Dear Dr. Chang,

Please submit your revised manuscript by Jun 17 2026 11:59PM. If you will need more time than this to complete your revisions, please reply to this message or contact the journal office at plosone@plos.org. When you're ready to submit your revision, log on to https://www.editorialmanager.com/pone/ and select the 'Submissions Needing Revision' folder to locate your manuscript file.

- A letter that responds to each point raised by the academic editor and reviewer(s). You should upload this letter as a separate file labeled 'Response to Reviewers'.
- A marked-up copy of your manuscript that highlights changes made to the original version. You should upload this as a separate file labeled 'Revised Manuscript with Track Changes'.
- An unmarked version of your revised paper without tracked changes. You should upload this as a separate file labeled 'Manuscript'.

If you would like to make changes to your financial disclosure, please include your updated statement in your cover letter. Guidelines for resubmitting your figure files are available below the reviewer comments at the end of this letter.

If applicable, we recommend that you deposit your laboratory protocols in protocols.io to enhance the reproducibility of your results. Protocols.io assigns your protocol its own identifier (DOI) so that it can be cited independently in the future. For instructions see: https://journals.plos.org/plosone/s/submission-guidelines#loc-laboratory-protocols. Additionally, PLOS ONE offers an option for publishing peer-reviewed Lab Protocol articles, which describe protocols hosted on protocols.io. Read more information on sharing protocols at https://plos.org/protocols?utm_medium=editorial-email&utm_source=authorletters&utm_campaign=protocols.

As the corresponding author, your ORCID iD is verified in the submission system and will appear in the published article. PLOS supports the use of ORCID, and we encourage all coauthors to register for an ORCID iD and use it as well. Please encourage your coauthors to verify their ORCID iD within the submission system before final acceptance, as unverified ORCID iDs will not appear in the published article. *Only*  the individual author can complete the verification step; PLOS staff *cannot*  verify ORCID iDs on behalf of authors.

We look forward to receiving your revised manuscript.

Kind regards,

Laura-Anne Marie Furlong

Academic Editor

PLOS One

Journal Requirements:

When submitting your revision, we need you to address these additional requirements.

1. Please ensure that your manuscript meets PLOS ONE's style requirements, including those for file naming. The PLOS ONE style templates can be found at https://journals.plos.org/plosone/s/file?id=wjVg/PLOSOne_formatting_sample_main_body.pdf and https://journals.plos.org/plosone/s/file?id=ba62/PLOSOne_formatting_sample_title_authors_affiliations.pdf

2. Please include your full ethics statement in the ‘Methods’ section of your manuscript file. In your statement, please include the full name of the IRB or ethics committee who approved or waived your study, as well as whether or not you obtained informed written or verbal consent. If consent was waived for your study, please include this information in your statement as well.

3. PLOS requires an ORCID iD for the corresponding author in Editorial Manager on papers submitted after December 6th, 2016. Please ensure that you have an ORCID iD and that it is validated in Editorial Manager. To do this, go to ‘Update my Information’ (in the upper left-hand corner of the main menu), and click on the Fetch/Validate link next to the ORCID field. This will take you to the ORCID site and allow you to create a new iD or authenticate a pre-existing iD in Editorial Manager.

4. We note that Figures 1 and 2 in your submission contain images which may be copyrighted. All PLOS content is published under the Creative Commons Attribution License (CC BY 4.0), which means that the manuscript, images, and Supporting Information files will be freely available online, and any third party is permitted to access, download, copy, distribute, and use these materials in any way, even commercially, with proper attribution. For more information, see our copyright guidelines: http://journals.plos.org/plosone/s/licenses-and-copyright.

We require you to either (1) present written permission from the copyright holder to publish these figures specifically under the CC BY 4.0 license, or (2) remove the figures from your submission:

1. You may seek permission from the original copyright holder of Figures 1 and 2 to publish the content specifically under the CC BY 4.0 license.

We recommend that you contact the original copyright holder with the Content Permission Form (http://journals.plos.org/plosone/s/file?id=7c09/content-permission-form.pdf) and the following text:

“I request permission for the open-access journal PLOS ONE to publish XXX under the Creative Commons Attribution License (CCAL) CC BY 4.0 (http://creativecommons.org/licenses/by/4.0/). Please be aware that this license allows unrestricted use and distribution, even commercially, by third parties. Please reply and provide explicit written permission to publish XXX under a CC BY license and complete the attached form.”

Please upload the completed Content Permission Form or other proof of granted permissions as an "Other" file with your submission.

In the figure caption of the copyrighted figure, please include the following text: “Reprinted from [ref] under a CC BY license, with permission from [name of publisher], original copyright [original copyright year].”

2. If you are unable to obtain permission from the original copyright holder to publish these figures under the CC BY 4.0 license or if the copyright holder’s requirements are incompatible with the CC BY 4.0 license, please either i) remove the figure or ii) supply a replacement figure that complies with the CC BY 4.0 license. Please check copyright information on all replacement figures and update the figure caption with source information. If applicable, please specify in the figure caption text when a figure is similar but not identical to the original image and is therefore for illustrative purposes only.

5. Please include captions for your Supporting Information files at the end of your manuscript, and update any in-text citations to match accordingly. Please see our Supporting Information guidelines for more information: http://journals.plos.org/plosone/s/supporting-information.

If the reviewer comments include a recommendation to cite specific previously published works, please review and evaluate these publications to determine whether they are relevant and should be cited. There is no requirement to cite these works unless the editor has indicated otherwise.

[Note: HTML markup is below. Please do not edit.]

Reviewers' comments:

Reviewer's Responses to Questions

**Comments to the Author**

1. Is the manuscript technically sound, and do the data support the conclusions?

Reviewer #1: Yes

Reviewer #2: No

**********

2. Has the statistical analysis been performed appropriately and rigorously?

Reviewer #1: Yes

Reviewer #2: Yes

**********

3. Have the authors made all data underlying the findings in their manuscript fully available?

The PLOS Data policy

Reviewer #1: Yes

Reviewer #2: No

**********

4. Is the manuscript presented in an intelligible fashion and written in standard English?

Reviewer #1: Yes

Reviewer #2: Yes

**********

Reviewer #1: I read this manuscript and I enjoyed the concept of the research which focuses on sports injuries and the wellbeing of tendons. There are many points to address before the manuscript is ready for publication.

Explain how the participants were made aware of this study and thus recruited in it.

It is important in a study like this to split volleyball players according to their playing position. A libero does not jump as much as attacking and blocking players, and setters don’t have the same jump and land biomechanics as hitters. Therefore, the authors must correct for playing position: liberos, setters, and attacking players. Also, it would be very interesting to see if the different players would show different patellar anatomies.

Please describe the breakdown between D1 and Olympic players. I noticed that the age range goes up to 34. was that specific athlete still playing professionally at that time, or have they retired and now play recreationally while maintaining the same workout regimen and play time?

It is not clear how many players never had PT, how many have a history but were asymptomatic when participated in the study. This is important to show.

Table1 needs to be split between no history of PT, history of PT but currently asymptomatic, and the currently symptomatic PT group.

Table1 Years of play: the range starts from 0...how is someone with 0 years of experience playing NCAA volleyball. Also, if they have 0 years of experience how are they suitable for inclusion in this study of elite players. Surely there should be a minimum number of years applied as inclusion criterion. How many players have less than 2 years experience?

Also, here it is important to split males from females especially that females in the US start playing volleyball at a younger age than males. Therefore, this will affect the average years of play.

Reviewer #2: The study is of interest and thorough, albeit carried out a long time ago (almost 20 years). Do the authors foresee any changes in the management of tendinopathy over the last 20 years affecting the relevance of the study today?

The authors set out their aim to understand how structural changes from jump-landings relate to anthropometrics and range of motion. Firstly, the authors replicate known results that tendinopathy correlates with increased tendon thickness and decreased PSFR. Because of this, the inclusion of participants both with and without tendinopathies is a clear confounding factor which makes it difficult to decouple whether the correlations observed relate to an individual's tendon morphology or their history of tendinopathy.

Throughout, very weak correlations are oversold based on the statistical significance of these weak correlations. I worry this will mislead readers as to the importance of these correlations. Regardless of correlation strength, publication of the results is certainly of interest to the field, but the weakness of these correlations should be made much clearer to readers throughout.

e.g. "A novel finding of our study is that distal tendon micromorphology, as indexed by the

PSFR, is significantly associated with hip and ankle joint ROM (Fig 3A)" - Figure 3A shows an incredibly low r^2 = 0.0484, and the tone of the text misrepresents this as an important correlation. Another example is in the conclusions: "Athletes with worse distal tendon morphology had reduced hip and ankle ROM, larger body mass, and greater mass concentration around the waist" - anyone can see from Figure 3 that this is hyperbole and does not reflect the reality of the results. Equally, I'm not convinced the title of the manuscript is supported.

A majority of the results are merely stated without graphical representation of the data and, given the above issues, it would be in the reader's interest to have visualisation of all stated results, either in figures in the main article or in supplementary material.

The introduction, methodology, and discussion sections mention a single leg squat test, the results of which don't appear to be included.

The acquired data is worthy of publication, however as the current conclusions are not supported by the data, I cannot recommend publication in its current state.

**********

what does this mean?). If published, this will include your full peer review and any attached files.

If you choose “no”, your identity will remain anonymous but your review may still be made public.

**Do you want your identity to be public for this peer review?**  For information about this choice, including consent withdrawal, please see our Privacy Policy

Reviewer #1: No

Reviewer #2: No

**********

[NOTE: If reviewer comments were submitted as an attachment file, they will be attached to this email and accessible via the submission site. Please log into your account, locate the manuscript record, and check for the action link "View Attachments". If this link does not appear, there are no attachment files.]

To ensure your figures meet our technical requirements, please review our figure guidelines: https://journals.plos.org/plosone/s/figures

You may also use PLOS’s free figure tool, NAAS, to help you prepare publication quality figures: https://journals.plos.org/plosone/s/figures#loc-tools-for-figure-preparation.

NAAS will assess whether your figures meet our technical requirements by comparing each figure against our figure specifications.

# Author comment: Author response to Decision Letter 1

**DOI:** 10.1371/journal.pone.0356562.r002

18 Jun 2026

We thank the reviewers for their thoughtful and constructive comments, which have helped us improve the clarity and rigor of our manuscript. We have provided a point-by-point response to each comment in the file "Response to Reviewers" and hope that the revisions adequately address the concerns raised. All changes to the manuscript are highlighted in the revised version for ease of reference.

Attachment Submitted filename: Response to Reviewers.docx

# Peer review: Decision Letter 1

**DOI:** 10.1371/journal.pone.0356562.r003

**Authors:** Laura-Anne Marie Furlong

4 Aug 2026

Relationship of patellar tendon micromorphology with body anthropometrics and lower-limb range of motion in volleyball athletes

PONE-D-26-03907R1

Dear Dr. Chang,

We’re pleased to inform you that your manuscript has been judged scientifically suitable for publication and will be formally accepted for publication once it meets all outstanding technical requirements.

Within one week, you’ll receive an e-mail detailing the required amendments. When these have been addressed, you’ll receive a formal acceptance letter and your manuscript will be scheduled for publication.

An invoice will be generated when your article is formally accepted. Please note, if your institution has a publishing partnership with PLOS and your article meets the relevant criteria, all or part of your publication costs will be covered. Please make sure your user information is up-to-date by logging into Editorial Manager at Editorial Manager® and clicking the ‘Update My Information' link at the top of the page. For questions related to billing, please contact billing support.

If your institution or institutions have a press office, please notify them about your upcoming paper to help maximize its impact. If they’ll be preparing press materials, please inform our press team as soon as possible -- no later than 48 hours after receiving the formal acceptance. Your manuscript will remain under strict press embargo until 2 pm Eastern Time on the date of publication. For more information, please contact onepress@plos.org.

Kind regards,

Laura-Anne Marie Furlong

Academic Editor

PLOS One

Additional Editor Comments (optional):

Reviewers' comments:

Reviewer's Responses to Questions

**Comments to the Author**

Reviewer #1: All comments have been addressed

Reviewer #2: All comments have been addressed

**********

2. Is the manuscript technically sound, and do the data support the conclusions?

Reviewer #1: Yes

Reviewer #2: Yes

**********

3. Has the statistical analysis been performed appropriately and rigorously?

Reviewer #1: Yes

Reviewer #2: Yes

**********

4. Have the authors made all data underlying the findings in their manuscript fully available?

The PLOS Data policy

Reviewer #1: Yes

Reviewer #2: Yes

**********

5. Is the manuscript presented in an intelligible fashion and written in standard English?

Reviewer #1: Yes

Reviewer #2: Yes

**********

Reviewer #1: The authors have addressed all my comments satisfactorily.

I enjoyed reading their work and wish them all the best in future projects.

I would be delighted to review more submissions by this group on this topic.

Reviewer #1.

Reviewer #2: The response of the authors is comprehensive, primarily including a rigorous reanalysis of the data. This has led to a greatly improved manuscript, and I am pleased to see this has also led to new and more in-depth results, discussions, and conclusions. The manuscript is an important reference for the relationships between body anthropometrics/ROMs and PSFR as a readout of collagen fibril organisation.

**********

what does this mean?). If published, this will include your full peer review and any attached files.

If you choose “no”, your identity will remain anonymous but your review may still be made public.

**Do you want your identity to be public for this peer review?**  For information about this choice, including consent withdrawal, please see our Privacy Policy

Reviewer #1: No

Reviewer #2: No

**********

# Editor report: Acceptance letter

**DOI:** 10.1371/journal.pone.0356562.r004

**Authors:** Laura-Anne Marie Furlong

PONE-D-26-03907R1

PLOS One

Dear Dr. Chang,

I'm pleased to inform you that your manuscript has been deemed suitable for publication in PLOS One. Congratulations! Your manuscript is now being handed over to our production team.

At this stage, our production department will prepare your paper for publication. This includes ensuring the following:

* All references, tables, and figures are properly cited

* All relevant supporting information is included in the manuscript submission,

* There are no issues that prevent the paper from being properly typeset

You will receive further instructions from the production team, including instructions on how to review your proof when it is ready. Please keep in mind that we are working through a large volume of accepted articles, so please give us a few days to review your paper and let you know the next and final steps.

Lastly, if your institution or institutions have a press office, please let them know about your upcoming paper now to help maximize its impact. If they'll be preparing press materials, please inform our press team within the next 48 hours. Your manuscript will remain under strict press embargo until 2 pm Eastern Time on the date of publication. For more information, please contact onepress@plos.org.

You will receive an invoice from PLOS for your publication fee after your manuscript has reached the completed accept phase. If you receive an email requesting payment before acceptance or for any other service, this may be a phishing scheme. Learn how to identify phishing emails and protect your accounts at https://explore.plos.org/phishing.

If we can help with anything else, please email us at customercare@plos.org.

Thank you for submitting your work to PLOS ONE and supporting open access.

Kind regards,

PLOS ONE Editorial Office Staff

on behalf of

Dr. Laura-Anne Marie Furlong

Academic Editor

PLOS One
